# Effects of adding mixed chicken and quail egg yolks to the cryodiluent on the quality of ram semen before and after cryopreservation

**DOI:** 10.3389/fvets.2022.1013533

**Published:** 2022-10-12

**Authors:** Ayman A. Swelum, Hani A. Ba-Awadh, Isiaka O. Olarinre, Islam M. Saadeldin, Abdullah N. Alowaimer

**Affiliations:** ^1^Department of Animal Production, College of Food and Agriculture Sciences, King Saud University, Riyadh, Saudi Arabia; ^2^Department of Theriogenology, Faculty of Veterinary Medicine, Zagazig University, Zagazig, Egypt; ^3^Department of Physiology, Faculty of Veterinary Medicine, Zagazig University, Zagazig, Egypt

**Keywords:** quail, chicken, preservation, egg yolk, fatty acids, ram, semen

## Abstract

The effects of adding mixed chicken and Japanese quail egg yolks (EYs) to the cryodiluent on the quality of ram semen before freezing and post-thawing were evaluated. Additionally, the composition of chicken and quail egg EYs and their mixture were analyzed for results explanation. The semen was collected from rams (*n* = 5) and extended with cryodiluent containing the EY of chicken, quail or their mixture (1:1). The extended semen was chilled slowly to 5 °C within 2 h and equilibrated for 2 h, before frozen on the liquid nitrogen vapor and cryopreserved at −196 °C. The straws were evaluated before freezing and post-thawing for sperm motility, vitality and abnormality besides plasma-membrane and DNA integrities. The moisture, ash, protein, and fatty acid (FA) contents of chicken EY, quail EY and their mixture were analyzed. Sperm vitality, plasma membrane integrity and DNA integrity before freezing were significantly (*P* < 0.05) higher in quail EY than chicken EY and mixed EYs cryodiluent. The chicken EY extender significantly improved the vitality, plasma membrane and DNA integrities of post-thawed ram semen in comparison with quail EY or mixed EYs extenders. While, the post-thawing sperm abnormalities was lower (*P* ≤ 0.05) in quail EY than chicken EY and mixed EYs cryodiluent. The post-thawing sperm motion kinetics parameters were higher in quail EY than chicken EY and mixed EYs cryodiluent. The highest percentages of moisture, ash, saturated fatty acids (SFAs) and monounsaturated fatty acids (MUFAs) were detected in quail EY had. While, the highest percentages of fat, protein and polyunsaturated fatty acids (PUFAs) were detected in chicken EY. In conclusion, using of chicken EY can improve total motility, vitality, plasma membrane integrity and DNA integrity of cryopreserved ram semen. While, using of quail EY can improve sperm abnormalities and kinetic motion parameters of cryopreserved ram semen. Mixing chicken and quail EYs added no value for post-thawing ram semen parameters.

## Introduction

Cryopreservation of semen is one of the essential technologies in animal research for enhancing productive and reproductive efficiencies (1). Semen cryopreservation helps to prolong sperm viability by slowing metabolism and preventing bacterial development, reducing the accumulation of metabolic byproducts (2–4). Cryopreservation of mammalian sperm is a complex procedure involving balancing several elements to produce the best results. Compared to other species, ram sperm has a low intramembrane cholesterol-to-phospholipid ratio. Therefore, ram sperms are more sensitive to cold-shock than sperms of other species (5).

Diluents, dilution, cooling, freezing, and thawing procedures all have a vital role in the success of ram semen cryopreservation (6, 7). The acrosome, nucleus, mitochondria, axoneme and plasma membrane, are all affected by rapid temperature fluctuations(cold shock) and the creation and dissolution of ice during the freezing-thawing process (3, 8). Cold shock damage occurs when sperm is rapidly cooled from 30 to 4 degrees Celsius (9). Temperature changes during cooling cause stress on sperm membranes, resulting in lipid phase shifts and a difference in the functional condition of sperm membranes. Therefore, semen must be diluted with a cryoprotectant extender to avoid sperm damage during freezing (10).

Egg yolk (EY) has a good effect as a non-permeable cryoprotectant at both cooling and sub-zero temperatures (11, 12). It has traditionally been used in commercial sperm extenders to protect the sperm membrane (13). The efficiency of using EY based diluents in the cryopreservation of animal semen including bull (14), camels (15), buck (16), fish (17) and boar (18) has been well demonstrated.

The mechanism by which EY protects sperm cells during cooling and freezing remains un fully understood. In 1939, the protective effects of EY on sperm during freezing were discovered for the first time (19). Low-density lipoproteins (LDLs) in the EY fraction have been identified as the active components (20). However, LDLs from sources other than EY did not provide the same protection level as EY (21, 22), which was likely because the LDL component of yolk contains numerous other substances, including proteins, which function in tandem to protect the sperm cells (20).

The chemical composition of the EYs of various avian species differs, particularly in terms of their cholesterol, fatty acid, and phospholipid contents (23, 24), which determine EY protection ability during cooling, freezing, and thawing (18). Conventionally, chicken EY has been used commonly as a non-permeable semen cryoprotectant, likely due to its ubiquitous availability (18). Nevertheless, EYs of other avian species improve the post-thaw quality of bull (25), ram (26–30), equine (31–35), buffalo (36–38) and boar (18) sperms.

Comparison among the efficacy of EYs of different avian species has started gaining momentum. Chicken and quail EYs have been reported to be better among many other avian species for cryopreservation of ram semen. Because of the relatively lower saturated fatty acids (SFA) ratio to polyunsaturated fatty acids (PUSFAs) in quail yolk (18), quail EY can result in better post-thaw quality or at least similar sperm protection as chicken EY in ram (28–30), jackass (31), buffalo (13) and Spanish Ibex (39). The information regarding the efficacy of using mixture of EYs of different avian species for semen cryopreservation is absent or scarce in the literature. To the best of our knowledge, the use of chicken and quail EYs mixture in cryodiluent for cryopreservation of ram semen has not been reported. Therefore, the objective of this study was to compare the efficacies of using chicken and quail EYs and their mixture in cryodiluent for cryopreservation of ram semen *via* semen evaluation before freezing and post-thawing. Additionally, the composition of chicken and quail egg EYs and their mixture were analyzed for results explanation.

## Materials and methods

### Management of animals

This experiment was performed in the autumn on seven mature, fertile Najdi rams aged 2–4 years with an average body condition score of 3 (0 = extremely thin, 5 = obese). At the experimental farm, Department of Animal Production, King Saud University, Riyadh, Saudi Arabia (latitude 24° 48′ N and longitude 46° 31′ E), rams were sheltered in a covered yard within an open-sided barn. The daily energy and protein requirements of rams were met with commercial mixed pellets (14.5 % crude protein and 2.78 Mcal metabolizable energy kg^−1^ dry matter, respectively). All rams were healthy and passed the breeding soundness evaluation test including semen evaluation using computer-assisted sperm analysis (CASA) (Sperm Class Analyzer^®^ version 4.0.0.5, Microptic S.L., Barcelona, Spain). The King Saud University Research Ethics Committee (REC) authorized the current project (Ethical Reference No: KSU-SE-21-33). This authorization is based on the advice of the Research Ethics Sub-Committee (minute number 7 and date 22/04/2021), as well as a suitable risk-to-benefit ratio and a study design that minimizes risks.

### Preparation of egg yolk extenders

Unless otherwise stated, all materials were obtained from Sigma (Sigma-Aldrich Corp., St. Louis, MO, USA). Tris-based buffer was prepared by dissolving 250 mM (3.028 g) Tris, 88.5 mM (1.78 g) citric acid, and 69.38 mM (1.25 g) fructose in 100 mL of distilled water (16). The buffer was then supplemented with 18 percent (v/v) EY and 8 percent (v/v) glycerol. Gentamicin (13.3 mg mL^−1^) was added. Three different diluents were prepared based on the type of egg yolk (EY): Tris chicken EY (C), Tris Japanese quail EY (Q), Tris chicken and Japanese quail EYs mixture (1:1) (CQ).

### Semen collection and evaluation

Semen samples were collected twice weekly from each ram using an artificial vagina. The color, density, volume, mass activity, sperm motility, and sperm cell concentration of each ejaculate were evaluated macroscopically and microscopically. Sperm motility and sperm cell concentration were automatically determined using a Sperm Class Analyzer^®^ (SCA^®^ version 4.0.0.5, Microptic S.L., Barcelona, Spain). The yellow and milky ejaculates that met the conventional parameters of 1.5 mL volume, 4 mass activity score, 80% sperm progressive motility, and 2 × 10^9^ sperm mL^−1^ concentration were selected for further processing. The ejaculates were pooled to eliminate individual ram variations. Seven pooled ejaculates were utilized in this study.

### Extension of semen and freezing

In a water bath, various types of diluents were used to dilute the semen gradually in a dilution rate 1:4 (semen: C, Q, or CQ). The diluted semen was gradually chilled within 2 h from 37 to 5 °C. The diluted cooled semen was loaded into 0.25 mL straws using a semiautomatic filling and sealing machine (minitube GmBH, Tiefenbach, Germany) and left at 5 °C for 2 h to allow for glycerol equilibration. After achieving equilibrium, the straws were frozen in liquid nitrogen vapor (5 cm above liquid nitrogen surface) for 15 min. The straws were then preserved at 196 °C *via* submerging in liquid nitrogen.

### Semen evaluation

The semen samples were evaluated after achieving glycerol equilibration (before freezing) and post-thawing. The frozen semen was examined after at least 2 weeks of cryopreservation. Thirty seconds were spent thawing the frozen straws in a 37 °C water bath. The semen evaluation was done mainly using SCA^®^ (version 4.0.0.5, Microptic S.L., Barcelona, Spain) which is computer-assisted sperm analysis (CASA) system for semen analysis allows the accurate, repetitive and automatic assessment of the following sperm parameters: motility, concentration, morphology, DNA fragmentation and vitality (16, 40). The details of CASA system was presented in Supplementary material.

### Sperm cell motility

SCA^®^ was used to evaluate sperm velocity in three ways (linear, curvilinear, and straight-line) for each sperm. The average pathway velocity (VAP), straight-line velocity (VSL), and curvilinear velocity (VCL) of sperm movement were measured. For evaluating progressiveness on a relative scale, the linearity (LIN = VSL/VCL), straightness (STR = VSL/VAP), and wobble (WOB = VAP/VCL) metrics were utilized and reported as a percentage. The amplitude of lateral head displacement (ALH) and beat cross frequency (BCF) were measured. The results were categorized according to velocity as rapid (VCL >75 μm/s), medium (45 < VCL < 75 μm/s), slow (10 < VCL < 45 μm/s) or static (VCL < 10 μm/s). The sperm presenting movement with a STR index ≥80% was considered progressive motile. The results were categorized as rapid progressive, slow progressive, non-progressive or static according to WHO 4th edition. The results were categorized as total progressive (summation of rapid and slow progressive), non-progressive or immotile according to WHO 5th edition.

### Sperm vitality

Using a FluoVit kit and protocol (Microptic S.L., Barcelona, Spain), the live/dead sperms were determined. Briefly, 10 μL of sperm sample was placed in a 1.5-ml Eppendorf tube, and 1 μL of warmed (37 °C) Hoechst trihydrochloride trihydrate stain was added. Five min were spent incubating this mixture at 37 °C. Then, 1 μL of warmed (37 °C) propidium iodide was added and stirred gently using a micropipette. Five min later, 10 μL of the stained sample was placed on a standard microscope slide with a cover glass and examined using fluorescence microscopy. A dead sperm displayed a fluorescent red hue, while a live sperm displayed a luminous blue hue. SCA vitality software was used to perform an automatic analysis. Each sample was tested with at least 200 sperms.

### Evaluation of sperm morphology and morphometry

The semen samples were placed on glass slides, and a conventional sperm smear was performed in duplicate and left to dry in the air. The slides containing sperm smears were placed vertically for 1–2 min on a staining tray with New Rapid SpermBlue^®^ fixative/stain (Microptic S.L., Barcelona, Spain), and the excess stain was drained. The slides were then dipped once in distilled water for approximately 3 sec, put at an angle of 60–80 degrees to remove excess fluid, and allowed to air dry. All of the sperm components (acrosome, head, midpiece, and main piece of tail) were stained with varying blue intensities. Sperm abnormalities were automatically determined and recorded as head length (m), head width (m), head area (m^2^), head perimeter (m), acrosome (percent), elongation, ellipticity, regularity, rugosity, midpiece width (m), midpiece area (m^2^), distance (m), and angle (°). The stained smears were examined with SCA^®^ abnormality software under a microscope at 600x magnification.

### Plasma-membrane integrity of sperm cells

A hypo-osmotic swelling test (HOST) was utilized as a supplemental test to the viability assessment technique to determine the functional integrity of the sperm plasma membrane. The experiment was carried out by incubating 10 μL of sperm with 100 μL of a 190 mOsm hypo-osmotic solution for 40 min at 37 °C in a 1.5-ml Eppendorf tube. On a warm slide, 100 μL of the sample was distributed with a cover slip. At least five distinct fields were utilized to count 200 sperms. The percentages of sperm with inflated and coiled tails were determined.

### DNA fragmentation of sperm cells

Halomax^®^ kit (Halotech, Madrid, Spain) was an *in vitro* diagnostic solution that allows the measurement of sperm DNA fragmentation for ram (Ovis aries). Halomax^®^ offers a better evaluation of semen quality compared to a traditional method which ignore the most important valuable parameter for a breeder, which is the quality of the genetic material that the sperm cell will transmit to the oocyte. Elevated sperm DNA fragmentation has a negative impact on pregnancy rates and litter size. The evaluation was done according to the manufacturer's instructions. Briefly, the samples of sperm were diluted to a concentration between 5 and 10 × 10^6^ mL^1^. The agarose gel from the kit was incubated at 90–100 °C for 5 min to fuse the agarose and then at 37 °C for 5 min in a water bath. Twenty-five microliters of the semen sample were added to the Eppendorf tube along with the gel and thoroughly mixed. Twenty microliters of the solution were deposited on a super-coated slide, then placed on a cool surface and covered with a 22x22-mm coverslip. Slides were refrigerated for 5 min at 4 °C to produce a micro-gel containing an implanted sperm. Carefully removing the coverslips, the slides were immersed for 7 min in the previously prepared acid solution (80 μL HCl in 10 mL of distilled water). The slides were then moved to a tray containing the lysing solution and incubated for 25 min, followed by rinsing with distilled water and dehydration in increasing ethanol concentrations for 2 min (70, 90, and 100 percent). The slides were stained with Giemsa dye or Wright stain, washed with tap water, and dried at 25 °C. Each slide was examined at 100 magnifications with a light microscope, and 200 sperms were scored. A sperm without fragmented DNA was placed in an agarose matrix and treated with lysing solutions to deproteinize the nucleus and generate the halos of dispersed DNA. The halos relate to the DNA loops loosely connected to the remaining nuclear structure (core). The sperm nuclei with fragmented DNA produce tiny or no halos of dispersed DNA, whereas the nuclei without fragmented DNA release DNA, forming large halos.

### Analysis of egg yolk

The moisture, ash, and protein content of three EYs group were evaluated. The fatty acids (FAs) of EY were quantified using gas chromatography. FA methyl esters were separated utilizing a GC-9A equipped with a flame ionization detector and a silica capillary column. At 35 mL min^−1^, nitrogen was used as a carrier gas. The pressure of hydrogen and air was established at 0.5 kg cm^−1^. The injector and detector were both kept at 250 degrees Celsius. A comparison of retention durations and standards was used to identify the FA methyl esters. The fatty acid composition was expressed as a percentage of the total FAs (41). For trace elements, EY samples from several avian species were digested in 69 percent nitric acid and 30 percent hydrogen peroxide utilizing a microwave acid digestion device (Ultrawave, Milestone, Monroe, CT, USA). With deionized water, the solutions were diluted to 25 mL. Cu (Copper), Zn (Zinc), Fe (Iron), Se (Selenium) and Mn (Manganese) contents in samples were determined using inductively coupled plasma–mass spectrometry (7700X ICP-MS, Agilent, Palo Alto, CA, USA). The concentrations of elements were given in milligrams per kilogram (ppm) of dry weight (42).

### Statistical analysis

All data were subjected to one-way analysis of variance (ANOVA) to compare the various types of extenders. The following model was used: “Y_jk_ = μ + A_k_ + e_jk_” where Y_jki_ is the target variable, μ is the overall mean, A_k_ is the fixed effect of the treatment (avian egg yolk extender), and e_jki_ is the residual error. Duncan's multiple range test was used to compare between the means of treatment to identify the statistically significant differences. The data was represented as mean ± standard error. Differences were considered significant at *P* < 0.05.

## Results

### Egg yolk analysis results

**Figure 1:**
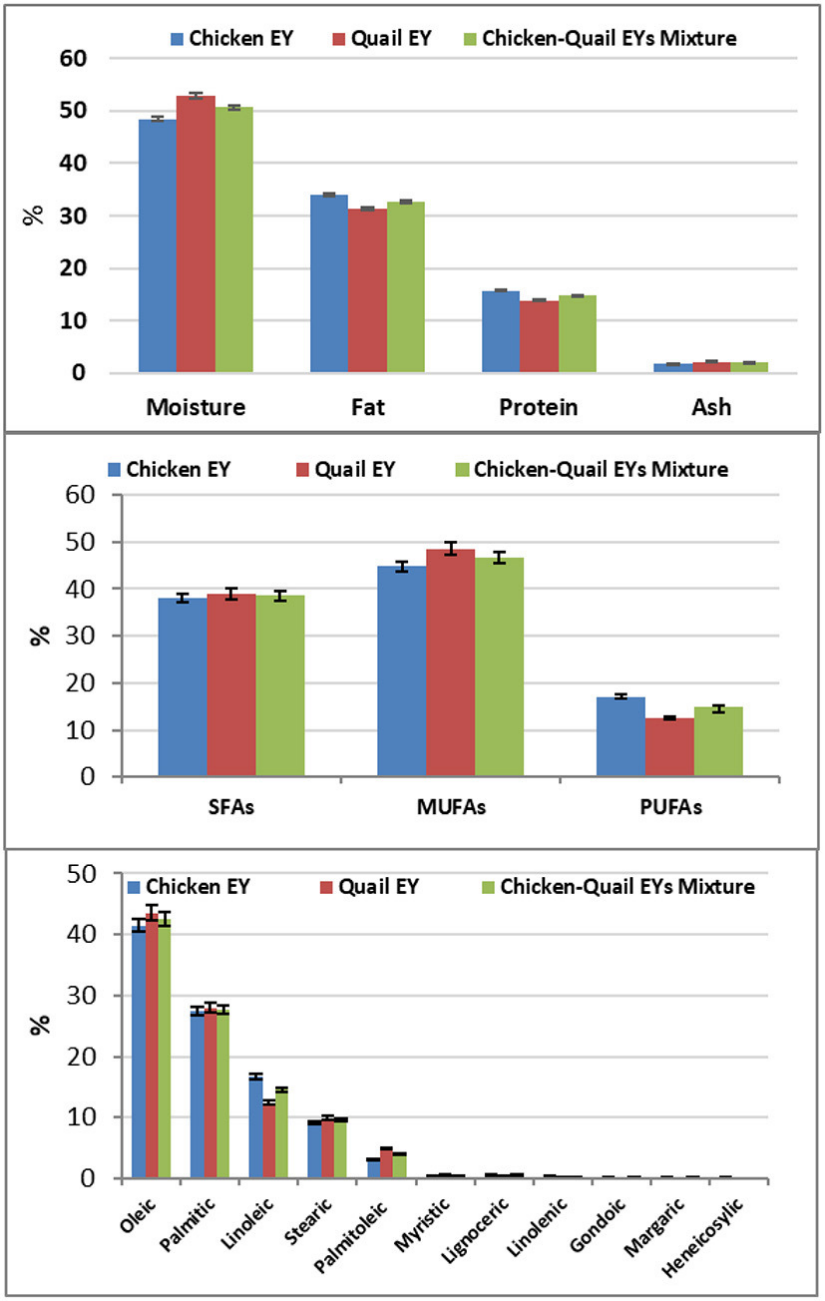
The component and fatty acids of chicken and Japanese quail egg yolk (EY) and their mixture (1:1).

**Figure 2:**
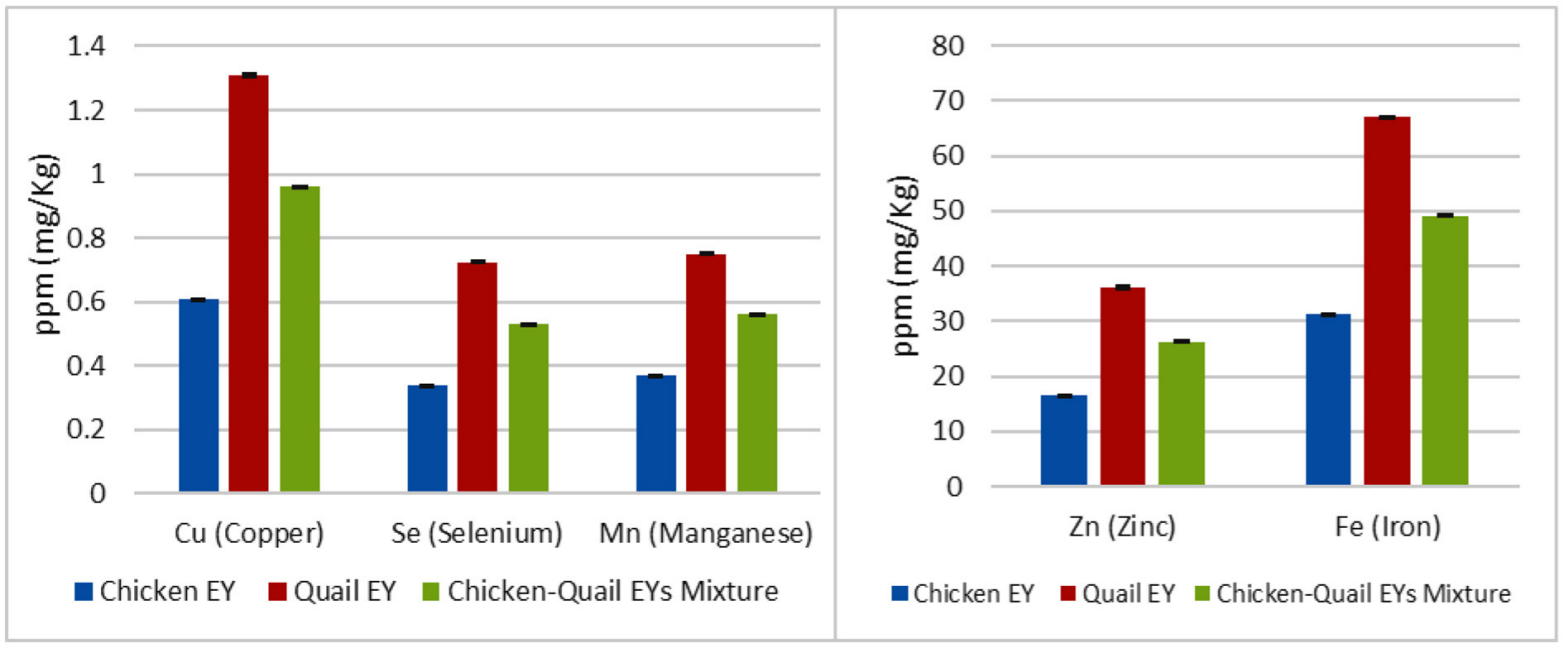
The Cu (Copper), Zn (Zinc), Fe (Iron), Se (Selenium) and Mn (Manganese) concentrations (mg/kg) in the chicken, Japanese quail egg yolk (EY) and their mixture.

Quail EY had higher percentages of moisture and ash; while, chicken EY had higher percentages of fat and protein. Quail EY had higher percentages of SFAs and MUFAs. In comparison, chicken EY had a higher percentage of PUFAs; quail EY had higher percentages of myristic, palmitoleic, and stearic. While, chicken EY had higher percentages of linoleic, linolenic, gondoic and lignoceric. No significant (*P* > 0.05) difference was observed between the three groups regarding percentages of oleic and palmitic FAs. Margaric and heneicosylic FAs were detected in chicken EY and were not detected in quail EY (Figure 1). Cu, Zn, Fe, Se and Mn concentrations were significantly higher in quail EY than in the mixture of chicken and quail EYs and chicken EY. Cu, Zn, Fe, Se and Mn concentrations were significantly higher in the chicken quail EYs mixture than in chicken EY (Figure 2).

### Results of semen analysis before freezing

**Figure 3:**
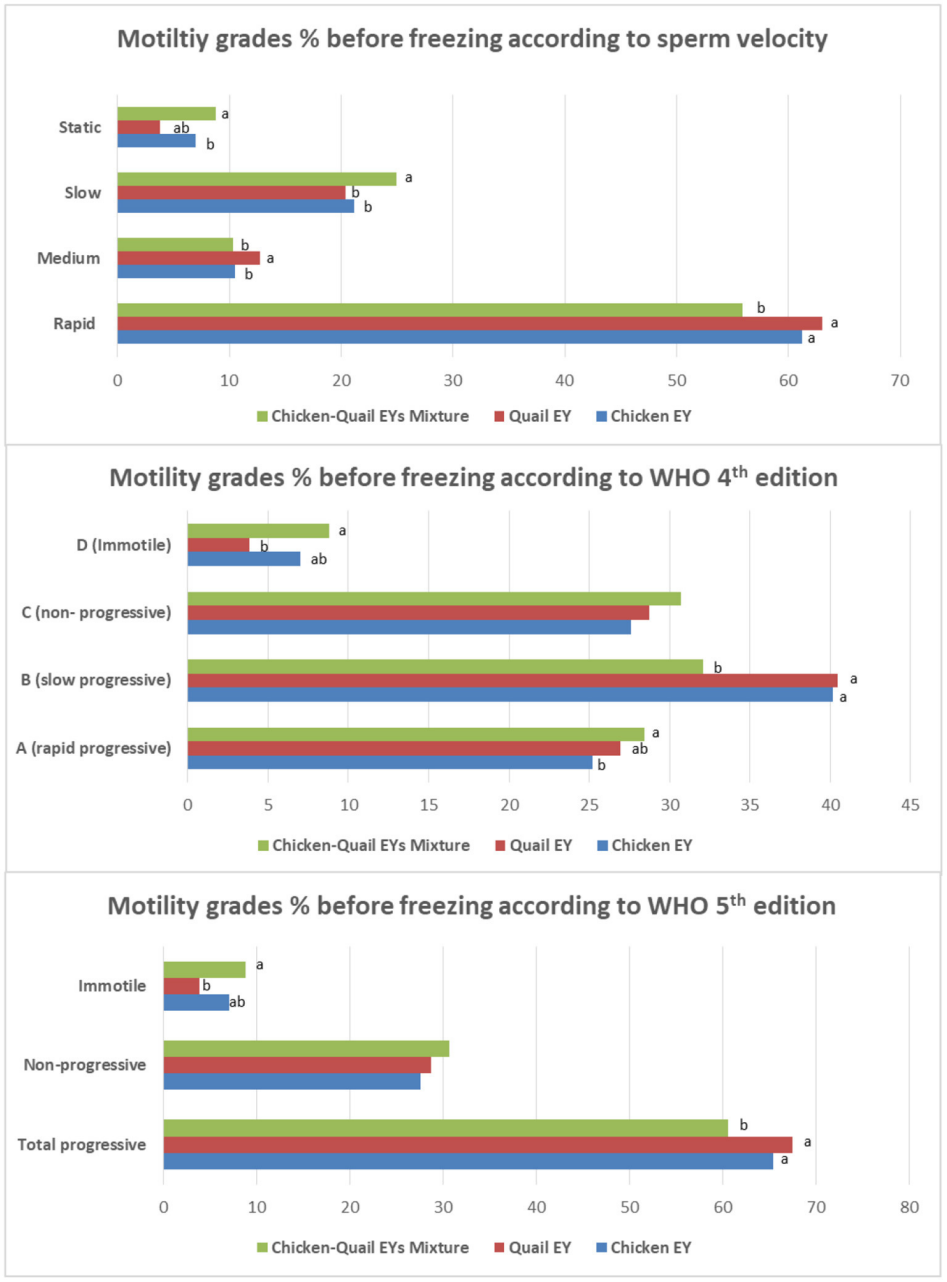
Effects of adding and combining egg yolks (EY) of chicken and/or quail to the Tris glycerol extender on motility grades of ram sperm before freezing. a,b,c, different letters indicate significant differences among the different egg yolks for each evaluated parameter; Progressive motility, Percentage of sperm presenting movement with a STR index ≥80% within the sample; Statics, VCL < 10 μm/s; Slow, 10 < VCL < 45 μm/s; Medium, 45 < VCL < 75 μm/s; Rapid, VCL >75 μm/s. In WHO 4th edition, the more details of progressive motility were added including slow or rapid progressive. While, in WHO 5th edition, the total progressive motility was presented as a one category regardless sperm velocity.

The rapid motility before freezing was significantly (*P* < 0.05) lower in CQ diluent than in the C and Q diluents. However, slow progressive motility before freezing was significantly (*P* < 0.05) lower in the CQ diluent than in C and Q diluents (Figure 3).

Total progressive motilities before freezing were significantly lower in CQ than C and Q diluents (Table 1). Total motility was significantly (*P* < 0.05) lower in the CQ diluent than in the Q diluent. Vitality, plasma membrane integrity and DNA integrity before freezing were significantly (*P* < 0.05) higher in Q diluents than in C and CQ diluents. Vitality and DNA integrity before freezing were significantly (*P* < 0.05) higher in C diluent than in CQ diluent. There was not significantly (*P* > 0.05) different between CQ and C diluents regarding plasma membrane integrity before freezing. However, the sperm abnormality before freezing was significantly (*P* < 0.05) lower in Q diluent than in C and CQ diluents.

**Table 1:** Effects of adding and combining egg yolks (EY) of chicken and/or quail to the Tris glycerol extender on motility, vitality, plasma membrane integrity, DNA integrity and abnormalities of ram sperm before freezing.

**Parameters**	**Chicken**	**Quail**	**Chicken-Quail**
	**EY**	**EY**	**EYs mixture**
Total progressive motility	65.37 ± 1.23^a^	67.41 ± 1.37^a^	60.51 ± 1.30^b^
Total motility	92.97 ± 1.39^ab^	96.15 ± 1.55^a^	91.20 ± 1.47^b^
Vitality	94.21 ± 0.62^b^	96.28 ± 0.69^a^	90.85 ± 0.65^c^
Plasma membrane integrity	94.45 ± 0.55^b^	97.07 ± 0.61^a^	93.10 ± 0.58^b^
DNA integrity	96.60 ± 0.54^b^	99.23 ± 0.54^a^	94.40 ± 0.54^c^
Sperm abnormality	11.60 ± 0.27^a^	7.63 ± 0.27^b^	12.03 ± 0.27^a^

^abc^Means ± Standard Error carrying different superscripts within the same row differed at P < 0.05.

The sperm VCL, VSL, VAP, WOB and ALH were significantly (*P* < 0.05) higher in C diluent than in Q and CQ diluents. The sperm VCL and ALH were significantly (*P* < 0.05) higher in Q diluent than in CQ diluent. While, the sperm VSL and WOB were significantly (*P* < 0.05) higher in CQ diluent than in Q diluent. LIN of sperm in C and CQ diluents was significantly (*P* < 0.05) higher than Q diluent. The STR of sperm was not significantly differed between three diluents. The BCF in Q diluent was significantly (*P* < 0.05) higher than C and CQ (Table 2).

**Table 2:** Effects of adding and combining egg yolks (EY) of chicken and/or quail to the Tris glycerol extender on motion kinetics parameters of ram sperm before freezing.

**Parameters**	**Chicken**	**Quail**	**Chicken-Quail**
	**EY**	**EY**	**EYs mixture**
VCL	98.79 ± 0.31^a^	90.46 ± 0.33^b^	87.98 ± 0.35^c^
VSL	52.90 ± 0.27^a^	48.44 ± 0.28^c^	50.73 ± 0.30^b^
VAP	75.88 ± 0.29^a^	69.00 ± 0.30^b^	69.87 ± 0.32^b^
LIN	50.42 ± 0.20^a^	49.40 ± 0.21^b^	50.77 ± 0.23^a^
STR	64.89 ± 0.20	64.25 ± 0.20	64.61 ± 0.22
WOB	73.66 ± 0.14^a^	72.33 ± 0.15^c^	72.87 ± 0.16^b^
ALH	4.27 ± 0.01^a^	4.06 ± 0.01^b^	3.63 ± 0.01^c^
BCF	4.27 ± 0.02^b^	4.46 ± 0.02^a^	3.89 ± 0.02^c^

VCL, curvilinear velocity (μms^−1^); VSL, straight-line velocity (μms^−1^); VAP, average pathway velocity (μms^−1^); LIN (%), linearity (VSL/VCL); STR (%), straightness (VSL/VAP); WOB (%), (VAP/VCL); ALH (μm), lateral head displacement; BCF(Hz), beat cross frequency.

^abc^Means ± Standard Error carrying different superscripts within the same row differed at P < 0.05.

The sperm head width in diluent CQ was higher than Q. The sperm head area and midpiece area before freezing were significantly (*P* < 0.05) higher in diluent CQ than C and Q. The percentage of sperm acrosome before freezing was significantly (*P* < 0.05) higher in C diluent than in Q diluent. No significant differences were observed between three diluents regarding head length, head perimeter, elongation, ellipticity, regularity, distance and angle (Table 3).

**Table 3:** Effects of adding and combining egg yolks (EY) of chicken and/or quail to the Tris glycerol extender on morphometry of ram sperm before freezing.

**Parameters**	**Chicken**	**Quail**	**Chicken-Quail**
	**EY**	**EY**	**EYs mixture**
Head length (μm)	9.98 ± 0.33	10.52 ± 0.33	10.86 ± 0.33
Head width (μm)	5.12 ± 0.12^ab^	4.93 ± 0.12^b^	5.33 ± 0.12^a^
Head area (μm^2^)	40.16 ± 1.21^b^	40.67 ± 1.21^b^	44.14 ± 1.21^a^
Head perimeter (μm)	26.42 ± 0.77	27.27 ± 0.77	28.59 ± 0.77
Acrosome (%)	86.09 ± 2.87^a^	77.07 ± 2.87^b^	80.16 ± 2.87^ab^
Elongation	0.32 ± 0.01	0.35 ± 0.01	0.34 ± 0.01
Ellipticity	1.94 ± 0.07	2.10 ± 0.07	2.03 ± 0.07
Regularity	0.99 ± 0.02	1.00 ± 0.02	1.03 ± 0.02
Rugosity	0.73 ± 0.02^a^	0.65 ± 0.02^b^	0.68 ± 0.02^ab^
Midpiece width (μm)	2.02 ± 0.15^a^	1.51 ± 0.15^b^	1.60 ± 0.15^b^
Midpiece area (μm^2^)	7.71 ± 0.52^a^	5.26 ± 0.52^b^	6.73 ± 0.52^a^
Distance (μm)	0.94 ± 0.25	0.37 ± 0.25	0.91 ± 0.25
Angle (°)	3.97 ± 3.37	11.27 ± 3.37	4.11 ± 3.37

^abc^Means ± Standard Error carrying different superscripts within the same row differed at P < 0.05.

## Results of semen analysis after freezing

**Figure 4:**
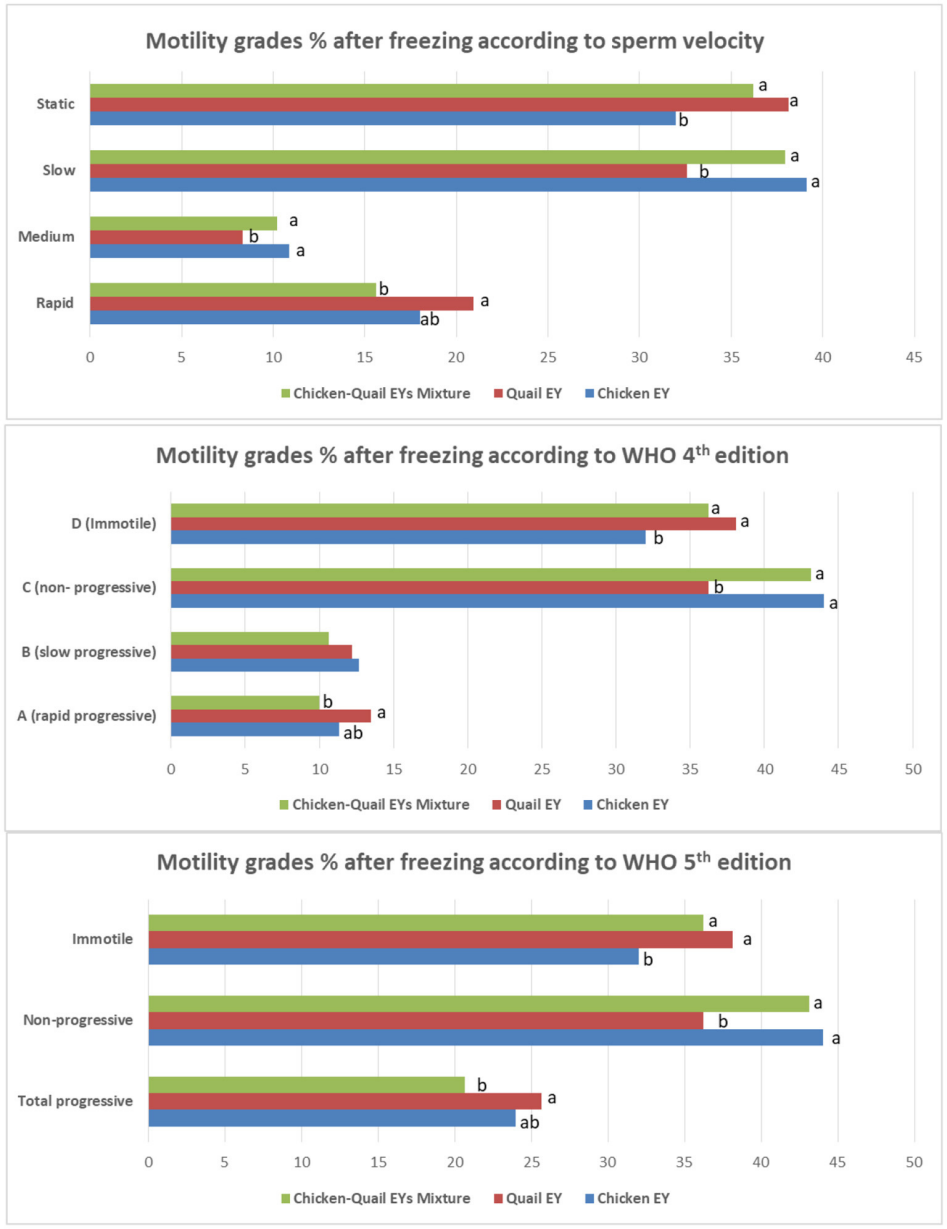
Effects of adding and combining egg yolks (EY) of chicken and/or quail to the Tris glycerol extender on motility grades of ram sperm post-thawing. a,b,c, different letters indicate significant differences among the different egg yolks for each evaluated parameter; Progressive motility, Percentage of sperm presenting movement with a STR index ≥80% within the sample; Statics, VCL < 10 μm/s; Slow, 10 < VCL < 45 μm/s; Medium, 45 < VCL < 75 μm/s; Rapid, VCL >75 μm/s. In WHO 4th edition, the more details of progressive motility were added including slow or rapid progressive. While, in WHO 5th edition, the total progressive motility was presented as a one category regardless sperm velocity.

The fast and rapid sperm motilities in the three diluents were not significantly (*P* > 0.05) different after freezing. There was no significant (*P* > 0.05) difference between the three diluents regarding medium and slow sperm motility. The static motility sperm in diluent C was significantly (*P* < 0.05) lower than in diluents in Q and CQ (Figure 4).

The total progressive motility of sperm after freezing was significantly higher in Q diluent than in CQ diluent (Table 4). The total motility, vitality, plasma membrane integrity and DNA integrity were significantly (*P* < 0.05) higher in C diluent than in Q and CQ diluents. The percentage of sperm abnormality was significantly (*P* < 0.05) lower in diluent Q than in C and CQ diluents. All sperm kinetic parameters were significantly higher in Q diluent than in C and CQ diluents (Table 5). All morphometric parameters except head length and regularity did not differ between the three diluents (Table 6).

**Table 4:** Effects of adding and combining egg yolks (EY) of chicken and/or quail to the Tris glycerol extender on motility, vitality, plasma membrane integrity, DNA integrity and abnormalities of ram sperm post-thawing.

**Parameters**	**Chicken**	**Quail**	**Chicken-Quail**
	**EY**	**EY**	**EYs mixture**
Total progressive motility	23.99 ± 1.13^ab^	25.65 ± 1.13^a^	20.63 ± 1.15^b^
Total motility	68.02 ± 1.28^a^	61.88 ± 1.28^b^	63.79 ± 1.30^b^
Vitality	69.19 ± 0.57^a^	63.63 ± 0.57^b^	64.98 ± 0.58^b^
Plasma membrane integrity	71.79 ± 0.51^a^	66.00 ± 0.51^c^	68.89 ± 0.51^b^
DNA integrity	86.60 ± 0.54^a^	82.97 ± 0.54^b^	81.33 ± 0.54^c^
Sperm abnormality	18.00 ± 0.27^a^	13.53 ± 0.27^b^	17.80 ± 0.27^a^

^abc^Means ± Standard Error carrying different superscripts within the same row differed at P < 0.05.

**Table 5:** Effects of adding and combining egg yolks (EY) of chicken and/or quail to the Tris glycerol extender on motion kinetics parameters of ram sperm post-thawing.

**Parameters**	**Chicken**	**Quail**	**Chicken-Quail**
	**EY**	**EY**	**EYs mixture**
VCL	49.27 ± 0.36^b^	54.78 ± 0.48^a^	48.70 ± 0.39^b^
VSL	28.46 ± 0.31^b^	33.11 ± 0.41^a^	27.46 ± 0.34^c^
VAP	37.51 ± 0.33^b^	42.73 ± 0.44^a^	36.68 ± 0.36^b^
LIN	47.32 ± 0.23^b^	50.64 ± 0.31^a^	45.43 ± 0.25^c^
STR	63.43 ± 0.23^b^	65.64 ± 0.30^a^	61.53 ± 0.25^c^
WOB	69.46 ± 0.16^b^	71.66 ± 0.21^a^	68.46 ± 0.18^c^
ALH	2.51 ± 0.01^b^	2.60 ± 0.02^a^	2.51 ± 0.02^b^
BCF	3.68 ± 0.02^b^	3.74 ± 0.02^a^	3.57 ± 0.02^c^

VCL, curvilinear velocity (μms^−1^); VSL, straight-line velocity (μms^−1^); VAP, average pathway velocity (μms^−1^); LIN (%), linearity (VSL/VCL); STR (%), straightness (VSL/VAP); WOB (%), (VAP/VCL); ALH (μm), lateral head displacement; BCF (Hz), beat cross frequency.

^abc^Means ± Standard Error (SE) carrying different superscripts within the same row differed at P < 0.05.

**Table 6:** Effects of adding and combining egg yolks (EY) of chicken and/or quail to the Tris glycerol extender on morphometry of ram sperm post-thawing.

**Parameters**	**Chicken**	**Quail**	**Chicken-Quail**
	**EY**	**EY**	**EYs mixture**
Head length (μm)	11.55 ± 0.51^a^	11.17 ± 0.53^b^	9.82 ± 0.52^b^
Head width (μm)	5.13 ± 0.14	5.11 ± 0.15	4.85 ± 0.14
Head area (μm^2^)	42.98 ± 1.68	42.62 ± 1.74	40.72 ± 1.71
Head perimeter (μm)	29.76 ± 1.08	29.24 ± 1.12	28.10 ± 1.10
Acrosome (%)	78.89 ± 4.10	78.40 ± 4.25	69.92 ± 4.17
Elongation	0.36 ± 0.02	0.37 ± 0.02	0.33 ± 0.02
Ellipticity	2.25 ± 0.11	2.21 ± 0.12	2.10 ± 0.12
Regularity	1.07 ± 0.03^a^	1.05 ± 0.03^a^	0.94 ± 0.03^b^
Rugosity	0.64 ± 0.03	0.64 ± 0.03	0.67 ± 0.03
Midpiece width (μm)	1.48 ± 0.29	1.73 ± 0.30	2.25 ± 0.30
Midpiece area (μm^2^)	4.94 ± 0.63	5.55 ± 0.65	4.58 ± 0.64
Distance (μm)	0.67 ± 0.29	1.10 ± 0.30	1.36 ± 0.29
Angle (°)	21.85 ± 6.57	10.54 ± 6.81	28.67 ± 6.68

^abc^Means ± Standard Error carrying different superscripts within the same row differed at P < 0.05.

## Discussion

Egg yolk and other non-penetrating cryoprotectants can protect sperm motility, vitality, and acrosome integrity during cooling and cryopreservation (5, 43, 44). Egg yolk is added to semen extenders to prevent early cold shock (16, 45). Egg yolk LDLs are the main active components (46). However, LDLs from sources other than EY did not provide the same protection as yolk (21, 22). This is likely because the LDL component of yolk contains various other chemicals, including proteins, that work in unison to protect sperm cells (46). Egg yolk LDL protects sperm membranes by binding and neutralizing seminal plasma proteins, which degrade sperm rapidly (47, 48). Therefore, LDL can improve sperm quality and fertility (43, 48). Due to its availability, chicken EY is the main avian EY used to cryopreserve mammalian sperm. However, EYs of other avian species succeed to cryopreserve semen of different animal species (25–38).

Our results after freezing revealed that the Q diluent was superior in the sperm abnormality, total progressive motility and all sperm kinetic parameters. While, C diluent was superior in the total motility, vitality, plasma membrane integrity and DNA integrity. Our findings contradict Kulaksiz et al. (28), who found that ram semen extended in other avian species EYs produced higher-quality sperm than ram sperm extended in chicken EY. Among chucker, chicken, goose, turkey, duck, and quail EYs utilized in ram semen cryodiluents, chucker EY was employed as a substitute to chicken EY and resulted in the highest sperm motility (54%) and vitality (59%) and the lowest sperm acrosomal abnormalities (28). Using of goose EY in ram cryodiluent was better than chicken and duck EYs; goose EY reduced acrosome defects (28). While, quail EY diluent improved sperm membrane integrity (28). Pigeon, duck, and turkey EYs were superior to chicken EYs for cryopreserving ram sperm in tris glycerol extender (26). Using turkey, ostrich, and duck EYs as ram semen diluent showed similar fertilization and embryonic development (27). In a recent experiment using chicken, quail, turkey, and duck EYs for ram semen cryopreservation, quail EY was an acceptable substitute for chicken EY because of it resulted in the lowest DNA fragmentation (8.8%) and the highest motility, sperm membrane integrity and vitality (29). The quail and turkey EYs extenders were superior to chicken and duck EYs in diluting epididymal semen after 4°C storage in Awassi rams (30). In ram and boar, chicken EY extender demonstrated better sperm parameters than quail EY extender (18, 28).

Our results are in agreement with the results of Trimeche, et al. (31), who found quail EY to be a viable replacement for chicken EY in frozen stallion sperm extenders. Using duck and chukar (Alectoris chukar) EYs to freeze stallion sperm improved post-thaw longevity and/or motility in comparison with chicken EY (32, 33).

In another experiment investigated the protective effects of EYs from five avian species (chicken, duck, goose, quail, and pigeon) on bull semen cryopreservation, the pigeon EY was suggested as a good alternative to chicken EY because it increased post-thawed vitality and progressive motility (25). For cryopreservation of buck sperm, chicken EY cannot be replaced by duck, goose, pigeon, quail, or turkey EY (16).

In another study comparing usage of quail and chicken EYs for cryopreservation of Spanish Ibex sperm, quail EY offered no advantages over chicken EY (39). The authors reported that sperm frozen with chicken EY resulted in higher motility, membrane integrity, and vitality in addition to lower abnormality than those frozen with quail EY. However, using quail EY improved acrosome intactness (39).

In buffalo bulls, duck EY extender has superior post-thaw sperm quality than chicken EY (45). In a similar experiment, quail EY at 5% and turkey EY at 10% were superior to chicken EY at 20% for *in vitro* post-thaw semen quality and *in vivo* buffalo fertility.

In the current study, the significant differences in evaluating parameters between quail and chicken EYs diluents and their mixture can be explained by the results of EY analysis which showed that quail EY had more SFAs and MUFAs and less PUFAs than chicken EY. Quail EY had more myristic, palmitoleic and stearic FAs and less linoleic, linolenic, gondoic, and lignoceric FAs than chicken EY. This explanation is in agreement with the finding of other researches who reported that the difference in post-thaw semen quality is due to the difference in the biochemical composition of EYs (18, 31). Varied avian EYs have different levels of phospholipids, FAs, and cholesterol (25, 28, 31). The significant difference in post-thaw ram semen quality cannot be explained only by FAs profile but also by other EY component as elements.

The lipid concentration and composition of the sperm plasma membrane determine its cryotolerance. Due to its lipid and FA contents, ovine sperm is particularly vulnerable to cold shock (49, 50). Ram sperm membrane has a low cholesterol/phospholipid ratio, low cardiolipin, phosphatidylserine, ethanolamine plasmalogen, and phosphatidylethanolamine concentrations, and a high content of phospholipids with PUFA, making them highly susceptible to cryodamage (49–53). Ram sperm and seminal plasma have 1.8 and 1.8% total lipids, respectively (54). SFA was 66.6 and 49.9% in seminal plasma and ram sperm, respectively (54). Butyric FA (C4:0) and Margaric FA (C16:0) are the most abundant SFA in ram sperm and seminal plasma (54). Docosahexaenoic acid was the highest abundant UFA in ram sperm and seminal plasma (54).

Our results revealed that all evaluating parameters significantly affected by freezing when comparing these parameters before and after freezing. Generally, post-thaw sperm motility, mitochondrial function, membrane integrity, and vitality decreased after cryopreservation and thawing. This drop in the post thawing parameters can be explained by the effect of cryopreservation on lipids. Cryopreservation of ram sperm alters choline glycerophospholipids with or without EY (55, 56). After cryopreservation, glycerophospholipids containing 22:6n-3 disappear (57). Cryopreservation impairs mitochondrial activity and damages mitochondria by removing two essential lipids (cardiolipin with 18:2n-6 and phosphatidylethanolamine with 20:4n-6) (57). After cryopreservation, sperm membrane lipids lose sterols, and sphingomyelin species with long chain PUFA decrease (57). These lipid alterations influence plasma membrane integrity, mitochondrial activity, and post-thawing characteristics. Any change in ram sperm lipid profile affects post-thawing sperm quality and cryopreservation outcomes (57). Therefore, preservation and protection of the lipid profile of ram sperm is the basis of any cryopreservation protocol to succeed. During cryopreservation, diluent lipids transfer to sperm membranes to protect it. Therefore, selection of FAs profile of diluent is important for obtaining best cryoprotectant effect.

FAs can be added to semen diluent to replace and/or replenish phospholipids lost during cryopreservation and thawing, increasing sperm osmotic and cryo-tolerances (58, 59). FAs improve sperm vitality, plasma membrane integrity, and livability during cryopreservation and reduces ice-crystal formation (60–62). Oleic and palmitic FA are the main sperm SFAs (63, 64). Palmitic or oleic FA increased bull sperm quality (65). Adding linolenic FA to BioXcell^®^ extender improved chilled and frozen bull sperm (60). Low oleic FA increased rooster semen quality during chilled storage (66). Boar sperm can utilize oleic and palmitic FA as ATP substrates *via* mitochondrial -oxidation (67). Therefore, oleic or palmitic FAs increased boar sperm quality during cold storage (67). Adding linolenic FA to buffalo diluent improved post-thaw semen quality and fertility (68). Adding n-3 linolenic FA can raise PUFA levels in the sperm membrane (69), improving membrane fluidity and sperm motility. Adding PUFAs to EY can extend shelf life of preserved ram semen (70). PUFAs increase the fluidity and cryo-tolerance of sperm membrane (71). As previously established, normal ram sperm morphology in chicken and fowl EYs diluents may be due to FA absorption (72).

Free FA toxicity affects sperm motility and aggregation (73). Linolenic FA (18:3) was more hazardous than linoleic FA (18:2) for human sperm (73). Oleic FA kills goat sperm (74). Palmitic FA (16:0) and stearic FA (18:0) were non-toxic to human sperm at 100 mg/dl.

Lipid peroxidation and profile alterations cause impaired sperm function (75, 76). During semen freeze-thaw, lipid peroxidation rises (77, 78). After abrupt temperature changes, mitochondria and sperm plasma membranes peroxidase (79, 80). Lipid peroxidation weakens sperm membranes and mitochondria (81, 82).

Disagreements in employing EYs from different avian species in semen extenders for cryopreservation of other animal species are related to differences in sperm membrane composition and EY components, which may result in species-specific interactions (39).

## Conclusions

In conclusion, mixing chicken and quail EYs added no value for post-thawing ram semen parameters. Chicken EY is superior in most post-thawing parameters, while quail EY is superior in sperm abnormalities and kinetic motion parameters. Therefore, chicken EY and quail EY can be used separately and without mixing in the ram semen cryodiluent. Further studies are needed to evaluate the fertility rate after using and mixing EYs from avian species.

## Data availability statement

The raw data supporting the conclusions of this article will be made available by the authors, without undue reservation.

## Ethics statement

The animal study was reviewed and approved by the King Saud University Research Ethics Committee (REC) authorized the current project (Ethical Reference No: KSU-SE-21-33). This authorization is based on the advice of the Research Ethics Sub-Committee (minute number 7 and date 22/04/2021), as well as a suitable risk-to-benefit ratio and a study design that minimizes risks.

## Author contributions

AS contributed to conceptualization, data curation, formal analysis, investigation, methodology, writing—original draft, review and editing. HB-A contributed to investigation and methodology. IO and IS contributed to writing—review and editing. AA contributed to funding acquisition, resources, software, supervision, validation, and visualization. All authors contributed to the article and approved the submitted version.

## Funding

This project was funded by the National Plan for Science, Technology and Innovation (MAARIFAH), King Abdulaziz City for Science and Technology, Kingdom of Saudi Arabia, Award Number (13-BIO2462-02).

## Conflict of interest

The authors declare that the research was conducted in the absence of any commercial or financial relationships that could be construed as a potential conflict of interest.

## Publisher's note

All claims expressed in this article are solely those of the authors and do not necessarily represent those of their affiliated organizations, or those of the publisher, the editors and the reviewers. Any product that may be evaluated in this article, or claim that may be made by its manufacturer, is not guaranteed or endorsed by the publisher.
