# Supplementary material for: Effects of adding mixed chicken and quail egg yolks to the cryodiluent on the quality of ram semen before and after cryopreservation
Source: Front Vet Sci. 2022 Oct 12;9:1013533. doi: 10.3389/fvets.2022.1013533 (PMC9596808; doi:10.3389/fvets.2022.1013533)
Supplement: Supplementary file 1 [file Data_Sheet_1.PDF]

MICROPTIC
AUTOMATIC DIAGNOSTIC SYSTEMS

SCA system

A- Software

SCA-PACK-VET-01: SCA Pack VET Edition (SCA-VET-01 + SCA-VET-03 + SCA-002)

SCA-VET-01: SCA® Motility and concentration - VET Edition

Module for the automatic analysis of the motility and concentration in animal sperm sample.

Main characteristics:

- **Multiple configurations** and the possibility to modify all the analysis parameters.
- Valid for **animal semen** only.
- Possibility of using **several specific digital cameras**, to analyze up to 100 frames per second and resolution up to 1024x1024 pixels.
- Automatic analysis per field in less than one second.
- Analysis in phase contrast or **fluorescence** (with FluoCount kit).

Possibility to capture up to 30 fields.

Permits to delete any field.

- Calculation of the **basic parameters**:
  - Sample concentration (M/ml and total ejaculate).
  - Number and percentage of the sample motility classified in the various types (progressive, non progressive, motile, statics, type a, type b, type c, type d, hyperactive).
- Calculation of **advanced parameters**: ○ Sample concentration (M/ml and total ejaculate) per motility type. ○ Average head area per type of velocity.
  - Allows to create groups/sort with peculiar characteristics.
- Calculation **average and by groups/types** of the kinetic parameters:
  - VCL: Curvilinear velocity. ○ VAP: Average path velocity. ○ VSL: Straight line velocity. ○ STR: Straightness index. ○ LIN: Linearity index. ○ WOB: Oscillation index.
  - ALH: Average Amplitude of the lateral head displacement.
  - BCF: Head beat frequency.
- Visualization of the trajectories for all the fields.
- Visualization of the **individual** motility characteristics of every spermatozoa and option to create a report.
- Possibility to add or eliminate trajectories.
- Possibility to **save sessions** for a later analysis.
- Possibility to **export images and videos**.
- Several types of reports with **images and graphics**, with the possibility to export them to Word, PDF, Excel, XML, TXT.
- Possibility to **customize the report**, adapting the information to the customer´s needs.
- Excel report includes a detailed list with all the parameters of every spermatozoa.
- Includes the **SCA® Sample Management**, that allows to retrieve the sample results to visualize them on the screen or print a wide range of reports (SQL Server database).
- Compatible with SCA® Viewer, program to visualize sessions in any computer.
- Compatible with SCA® Capture, SCA® drops, SCA® dataShare and SCA® Stage Controller.

MICROPTIC S.L
Viladomat, 321 - 6º 4ª
08029 Barcelona (Spain)
Tel: +34 93 419 29 10 - Fax: +34 93 419 94 26
Web: www.micropticsl.com - E-mail: micropticsl@micropticsl.com
TÜVRheinland CERTIFIED
Management System
ISO 9001:2008
ISO 13485:2003
www.tuv.com
ID 9105064132

SCA-VET-03: SCA® Morphology - VET Edition

Module for the automatic analysis of the sperm morphology and morphometry of species having oval spermatozoa.

Main characteristics:

- **Multiple configuration** and the possibility to modify all analysis parameters.
- Possibility to create personal matrix chart for the analysis.
- Valid for **animal semen samples having oval spermatozoa**.
- Allows the use **several specific digital cameras**, with resolution up to 1024x1024 pixels.
- Automatic detection and analysis of all the spermatozoa in the field (head, acrosome, midpiece and tail).
- Allows the capture up to 200 fields.

Several staining kits allowed.

Calculation of **basic parameters**:

- o Percentage of normal and abnormal spermatozoa. o Percentage of spermatozoa with abnormal head, midpiece, tail and cytoplasmic droplet.
- o Teratozoospermy index.

- Calculation of **advanced parameters**:
  - o Morphometry analysis (average and standard deviation)
    - ▪ Head: Length, width, area, perimeter, elongation, ellipticity, rugosity, regularity, percentage of acrosome, grey level.
    - ▪ Midpiece: Width, area, insertion distance, angle. ▪ Tail: Length.
  - o Percentage of each morphological type (It is possible to modify the description) ▪ Head: Micro, macro, elongated, pyriform, round, amorphous, normal acrosome.
    - ▪ Midpiece: Width, asymmetric, angulated.
    - ▪ Tail: Short, bent, coiled, multiple, without.
- Visualization of the analysis masks and the original image.
- Visualization of each spermatozoa **individually** and possibility to print these results in an individual report.
- Possibility to add or eliminate spermatozoa.
- Manual selection of tail anomalies.
- Permits to **save a session** for a later analysis.
- Possibility to **export images**.
- Several reports are provided with **graphics and images**. Possibility to export the reports to Word, PDF, Excel, XML, TXT.
- Possibility to **customize the report**, adapting the information to the customer´s needs.
- Excel report with the list of morphology parameters of each spermatozoa.
- Includes **SCA® Sample Management** , to retrieve the sample results at any time, and visualize them on the screen or print a report (SQL server database).
- Compatible with SCA® Viewer, that allows displaying sessions in any computer.
- Compatible with SCA® Capture, SCA® dataShare and SCA® Stage Controller.

SCA-002: SCA® Manual counter

Module developed to analyse, in a manual way, any sample that can be visualized with the digital camera used.

MICROPTIC S.L
Viladomat, 321 - 6º 4ª
08029 Barcelona (Spain)
Tel: +34 93 419 29 10 - Fax: +34 93 419 94 26
Web: www.micropticsl.com - E-mail: micropticsl@micropticsl.com

TÜVRheinland CERTIFIED Management System ISO 9001:2008 ISO 13485:2003 www.tuv.com ID 9105064132

Main characteristics:

- Visualization of the sample on the screen.
- Permits to save into the database the obtained results.
- Allows the creation of customized counters (for any sample).
- Configuration of the counter keys.
- Results report with pictures that can be manually captured.
- Camera controls to modify the colours, brightness and contrast of the displayed image.

SCA-VET-05: SCA® DNA Fragmentation - VET Edition

Module for the automatic analysis of the DNA fragmentation in an animal semen sample.
Main characteristics:

Multiple configurations, and possibility to modify al the analysis parameters.
Valid for **animal semen** only.

- Allows to use **several specific digital cameras**, with resolution up to 1024x1024 pixels.
- Automatic selection and analysis of all the spermatozoa in the field.
- Analysis under brightfield or under **fluorescence** (using Halomax kit).
- Allows to capture up to 200 fields.
- Allows to delete any field.
- Calculation of the **basic parameters**: ○ Number and percentage of fragmented and on fragmented spermatozoa.
- Calculation of **advanced parameters**:
  - ○ Number and percentage for spermatozoa with: Big halo, medium halo, small halo, without halo or degraded.
- Visualization of the analysis mask superimposed to the original image.
- **Individual** visualization of each analysed spermatozoon and possibility of individual report.
- Allows to add or delete spermatozoa.
- Allows to **save sessions** for a later analysis.
- Allows to **export images**.
- Several reports with **graphics and images** that can be exported to Word, PDF, Excel, XML and TXT.
- Possibility to **customize the report**, adapting the information to the customer´s needs.
- Excel report with all the listed parameters of DNA fragmentation.
- Includes **SCA® Sample Management**, to retrieve the sample results at any time, and visualize them on the screen or print a report (SQL Server database).
- Compatible with SCA® Viewer, that allows to display sessions in any computer.
  Compatible with SCA® Capture, SCA® dataShare and SCA® Stage Controller.

SCA-VET-02: SCA® Vitality - VET Edition

Module for the automatic analysis of the vitality in animal semen samples under fluorescence.
Main characteristics:

- Multiple configurations and the option to modify all the analysis parameters.
- Valid for animal semen samples only.
- Allows to use several specific digital cameras, with resolution up to 1024x1024 pixels.

• Automatic selection and analysis of all the spermatozoa in the field.
• Analysis under fluorescence (for example with FluoVit kit).
• Obtaining this module and SCA® Motility and concentration, vitality can be analysed at the same time as motility and concentration, and under fluorescence.
• Allows to capture up to 30 different fields.
• Allows to eliminate any field.
• Calculation of basic parameters: o Number and percentage of live and dead spermatozoa.
• Visualization of the analysis mask superimposed to the original image.
• Possibility to add or eliminate spermatozoa.
• Allows to save sessions for a later analysis.
• Allows to export images.
• Several reports are produced with images and graphics, that can be exported to Word, PDF, Excel, XML, TXT.
• Possibility to customize the report, adapting the information to the customer´s needs.
• Includes SCA® Sample Management, to retrieve the sample results at any time, and visualize them on the screen or print a report (SQL Server database).
• Compatible with SCA® Viewer, that allows to display sessions in any computer.
• Compatible with SCA® Capture, SCA® dataShare and SCA® Stage Controller.

B- Hardware

CU-SCA: SCA° control unit (CU-DESKTOP-S + CB-ACA-780 + GE-PCIE + FS10J-2)

SCA° control unit containing:

CU-DESKTOP-S: Desktop computer

HP desktop computer main characteristics:

- Intel Core i5 processor
- 500GB hard drive
- 4GB RAM memory
- Intel HD graphic card
- DVD writer
- Windows 8 Pro (Windows 7 Pro available)

(Monitor not included)

CB-ACA-780: Basler camera Ace ACA780-75GC Main features:

- Resolution: 782x582 pixels
- Frame rate: 75 fps
- Capture: Black/white and color
- Interface: Gigabit Ethernet
- Lens mount: C-mount
- Weight: 90g
- Sensor technology: Progressive Scan CCD
- Sensor size: ½ inch
- Sensor type: CCD

GE-PCIE: Gigabit Ethernet PCI-Express card

Gigabit Ethernet PCI-Express card for desktop computer.

FS10J-2: Quick image capture USB foot pedal

Viladomat, 321 - 6º 4ª
08029 Barcelona (Spain)
Tel: +34 93 419 29 10 - Fax: +34 93 419 94 26
Web: www.micropticsl.com - E-mail: micropticsl@micropticsl.com
TÜVRheinland CERTIFIED Management System ISO 9001:2008 ISO 13485:2003 www.tuv.com ID 9105064132

Quick image capture USB foot pedal. USB connection.

NK-E200-LED: Nikon E200-LED microscope Nikon E200-LED microscope including:

- MCA76200 Eclipse LED basic unit
- MBF41300 Connection cable
- MCB73100 Trinocular tube
- MBB73520 TV tube
- MQD42000 CCD Camera Adaptor C-ISO mount (1x) for digital camera
- MCK70100 CFI ocular E 10X (x2)
- MBL73100 Phase contrast condenser AN 0,9 (turret type)
- MBN11200 GIF Filter
- MBN11710 NCB-11 Filter
- MXA20233 Immersion oil (8 ml)

MAK85005: Centering telescope C-CT Nikon

Nikon centering telescope with Bertrand lens for condenser phase rings alignment.

MRP00202: Nikon 20x CFI achromatic objective (brightfield)

Nikon CFI Achromatic objective LWD 20x A A.N. 0,4 W.D. 3,9mm. 60mm parfocal distance, 25mm mount.

MRP00602: Nikon 60x CFI achromatic objective

Nikon 60x CFI achromatic objective A.N. 0,80 W.D. 0,3 mm. 60mm parfocal distance, 25 mm mount.

MRP71900: Nikon E200 100x oil CFI achromatic objective (brightfield)

Nikon 100x oil CFI achromatic objective A.N. 1,25 W.D. 0,23mm with spring protection. Immersion in oil. 60mm parfocal distance, 25mm mount.

MRP90100: Nikon 10x Ph- CFI achromatic objective (phase contrast)

Nikon 10x Ph- CFI achromatic BM objective AN 0.25 WD 6.1mm. Negative phase contrast.

MBE41200: EPI-Fl C-FL filter block UV-2A

Nikon EPI-Fluorescence C-FL filter block UV-2A.

MBE94310: Nikon EPI-Fluorescence module CI-FL

EPI-Fluorescence module CI-FL for Nikon microscope.

UV-LED-NIK: UV-LED Prior with Nikon adapter

Ultra Violet LED light source Prior for Nikon fluorescence microscopes.

TÜVRheinland CERTIFIED
Management System
ISO 9001:2008
ISO 13485:2003
www.tuv.com
ID 9105064132

HS-DUO: Heating stage double

Modification of the original stage to add the heating attachments, and temperature controller with additional heating plate above. The stages are built from the original standard stage that comes with the microscope: Nikon and Olympus.

MICROPTIC S.L
Viladomat, 321 - 6º 4ª
08029 Barcelona (Spain)
Tel: +34 93 419 29 10 - Fax: +34 93 419 94 26
Web: www.micropticsl.com - E-mail: micropticsl@micropticsl.com
TÜVRheinland CERTIFIED
Management System
ISO 9001:2008
ISO 13485:2003
www.tuv.com
ID 9105064132
